# Supplementary figures and images for: Potential benefits of advanced chelate-based trace minerals in improving bone mineralization, antioxidant status, immunity, and gene expression modulation in heat-stressed broilers
Source: PLoS One. 2024 Oct 2;19(10):e0311083. doi: 10.1371/journal.pone.0311083 (PMC11446444; doi:10.1371/journal.pone.0311083)

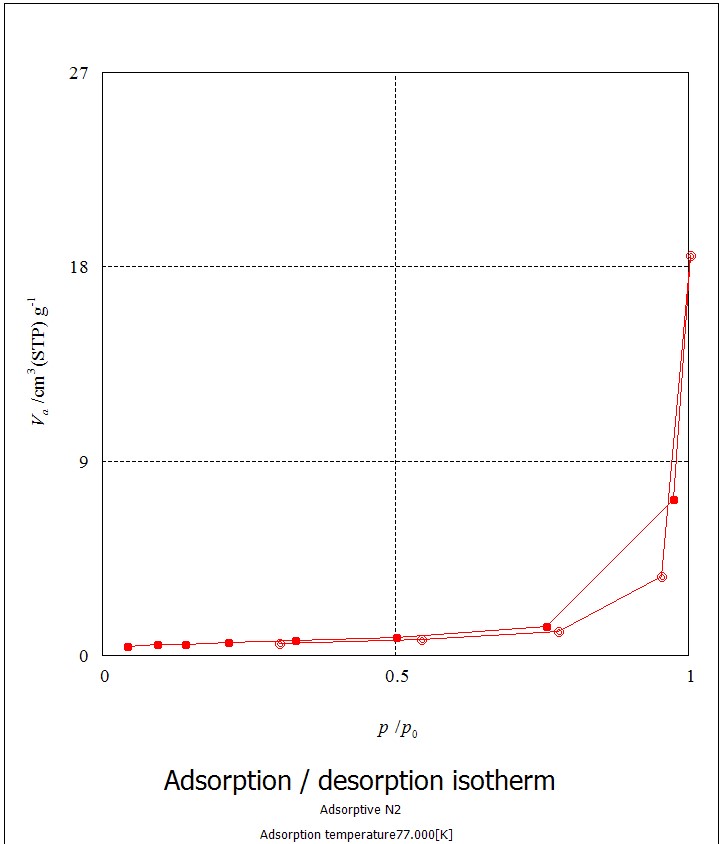


**Fig S1.** The BET analysis of advanced chelates crystallinity used in this study

Supplement: S1 Fig — (DOCX) [file pone.0311083.s001.docx]
