# Supplementary material for: Potential benefits of advanced chelate-based trace minerals in improving bone mineralization, antioxidant status, immunity, and gene expression modulation in heat-stressed broilers
Source: PLoS One. 2024 Oct 2;19(10):e0311083. doi: 10.1371/journal.pone.0311083 (PMC11446444; doi:10.1371/journal.pone.0311083)
Supplement: S1 Table — (DOCX) [file pone.0311083.s002.docx]

**Table S1.** Supplemental level and analyzed content of trace minerals in experimental diets (mg/kg)

|  |  | Experimental treatments^1^ | | | | |  |
| --- | --- | --- | --- | --- | --- | --- | --- |
| Item |  | ITM | ACTM50 | ACTM100 | ITM+ACTM12.5 | ITM+ACTM25 | ITM125 |
| Supplemental level |  |  |  |  |  |  |  |
| Iron |  | 80 | 40 | 80 | 90 | 100 | 100 |
| Zinc |  | 90 | 45 | 90 | 101 | 113 | 113 |
| Manganese |  | 100 | 50 | 100 | 113 | 125 | 125 |
| Copper |  | 16 | 8 | 16 | 18 | 20 | 20 |
| Selenium |  | 0.30 | 0.15 | 0.30 | 0.34 | 0.38 | 0.38 |
| Iodine |  | 1.20 | 0.60 | 1.20 | 1.35 | 1.50 | 1.50 |
| Chromium |  | 0.100 | 0.050 | 0.100 | 0.113 | 0.125 | 0.125 |
| Analyzed mineral content | | |  |  |  |  |  |
| d 1 to 10 |  |  |  |  |  |  |  |
| Iron |  | 149 | 112 | 151 | 163 | 169 | 174 |
| Zinc |  | 123 | 84 | 125 | 131 | 144 | 146 |
| Manganese |  | 136 | 89 | 136 | 151 | 163 | 155 |
| Copper |  | 21.2 | 12.5 | 20.2 | 22.0 | 25.3 | 24.2 |
| Selenium |  | 0.39 | 0.30 | 0.42 | 0.45 | 0.46 | 0.51 |
| Iodine |  | 1.16 | 0.68 | 1.19 | 1.27 | 1.45 | 1.38 |
| Chromium |  | 0.091 | 0.052 | 0.098 | 0.104 | 0.117 | 0.107 |
| d 10 to 24 |  |  |  |  |  |  |  |
| Iron |  | 151 | 114 | 142 | 153 | 167 | 168 |
| Zinc |  | 121 | 82 | 127 | 134 | 145 | 142 |
| Manganese |  | 138 | 88 | 140 | 149 | 163 | 158 |
| Copper |  | 20.7 | 12.8 | 21.0 | 22.4 | 25.7 | 26.2 |
| Selenium |  | 0.39 | 0.28 | 0.41 | 0.45 | 0.49 | 0.47 |
| Iodine |  | 1.24 | 0.76 | 1.22 | 1.34 | 1.48 | 1.51 |
| Chromium |  | 0.092 | 0.055 | 0.107 | 0.111 | 0.116 | 0.110 |
| d 24 to 42 |  |  |  |  |  |  |  |
| Iron |  | 148 | 112 | 143 | 155 | 163 | 169 |
| Zinc |  | 119 | 81 | 126 | 134 | 143 | 138 |
| Manganese |  | 134 | 84 | 139 | 150 | 162 | 155 |
| Copper |  | 21.0 | 13.8 | 21.3 | 22.8 | 25.7 | 26.0 |
| Selenium |  | 0.40 | 0.31 | 0.39 | 0.42 | 0.47 | 0.51 |
| Iodine |  | 1.25 | 0.74 | 1.27 | 1.38 | 1.50 | 1.46 |
| Chromium |  | 0.096 | 0.054 | 0.103 | 0.109 | 0.118 | 0.113 |

^1^ITM, refer to 3 treatments [TNC (thermoneutral group), HSC (challenged with heat stress)] containing commercially recommended levels of inorganic trace mineral (ITM); ACTM50, advanced chelate technology-based trace minerals (ACTM) match to 50% of the ITM; ACTM100, ACTM equivalent to ITM; ITM+ACTM12.5, ACTM added to the ITM diet at the level of 12.5% above the commercially recommended levels for TM; ITM+ACTM25, ACTM added to the ITM at the level of 25% above the recommended levels for TM. ITM125, containing 125% commercially recommended levels for ITM.
